# Prognostic value of postoperative decrease of albumin (ΔAlb) in combination with GLIM-defined malnutrition for the prediction of postoperative outcomes in rectal cancer patients with normal preoperative albumin levels

**DOI:** 10.3389/fnut.2025.1542581

**Published:** 2025-05-13

**Authors:** Chong-Jun Zhou, Ke-Yu Ling, Jun Fang, Meng-Fan Ye, Meng-Li Liu, Jia-Can Chen, Chen-Guo Zheng, Shao-Tang Li

**Affiliations:** ^1^Department of Anorectal Surgery, The Second Affiliated Hospital and Yuying Children’s Hospital of Wenzhou Medical University, Wenzhou, Zhejiang, China; ^2^Department of Anorectal Surgery, The Third People’s Hospital of Cangnan County, Wenzhou, Zhejiang, China; ^3^National Key Clinical Specialty (General Surgery), The First Affiliated Hospital of Wenzhou Medical University, Wenzhou, Zhejiang, China; ^4^School of Laboratory Medicine and Life Science, Wenzhou Medical University, Wenzhou, Zhejiang, China

**Keywords:** rectal cancer, postoperative complications, serum albumin, ΔAlb, Malnutrition

## Abstract

**Objective:**

This study aims to explore the prognostic value of ΔAlb in combination with malnutrition for postoperative outcomes in rectal cancer patients with normal preoperative albumin levels.

**Methods:**

We conducted a retrospective study of patients undergoing proctectomy for rectal cancer at our department between January 2013 and April 2019. Malnutrition was defined according to the Global Leadership Initiative on Malnutrition (GLIM) criteria. A receiver operating characteristic curve analysis was used to determine the cut-off values for ΔAlb. Univariate and multivariate analyses evaluating the risk factors for postoperative complications and ΔAlb were performed.

**Results:**

A total of 526 patients were enrolled in this study. ∆Alb was significantly associated with postoperative complications in patients with normal preoperative albumin levels (AUC = 0.651, *p* < 0.001), but was not in patients with hypoalbuminemia (*p* = 0.808). The optimal cut-off value was established at 16%. ∆ALB ≥ 16% and malnutrition were both independent risk factors for postoperative complications with an odds ratio (OR) of 2.179 and 1.730, respectively. When combined then together, the OR would reach to 3.779. On the other hand, low muscle mass (OR = 2.058, *p* < 0.001), tumor location in the lower third (OR = 2.909, *p* < 0.001), and surgical duration ≥ 180 min (OR = 1.659, *p* = 0.01) were identified as independent risk factors associated with ∆ALB.

**Conclusion:**

ΔAlb in combination with GLIM-defined malnutrition would enhance the predictive value for postoperative outcomes in rectal cancer patients with normal preoperative albumin levels, and it is necessary to conduct a nutritional assessment for then.

## 1 Introduction

Despite advancements in minimally invasive surgical techniques and perioperative care, postoperative complications following colorectal cancer surgery remain a major impediment, cause prolonged in-hospital stays, increase hospital costs, even threaten survival, especially in rectal cancer (1–3). Therefore, early identification of risk factors for postoperative complications is of paramount importance. Recently, postoperative decrease of albumin (ΔAlb), a marker reflecting the extent of surgical trauma, was widely reported as an early predict factor for complications after major abdominal surgery (4). Due to the heterogeneity in ΔAlb thresholds, various types of surgery and the absence of high-quality studies, it remains a challenge to apply ΔAlb in clinical practice.

Traditionally, albumin is regarded as a nutritional marker, and numerous postoperative complications are associated with hypoalbuminemia (5). Therefore, more attention has been paid to hypoproteinemia and treated with nutritional interventions, which neglecting the malnourished patients with normal albumin levels. In fact, the idea that albumin signifies nutritional condition is arbitrary and inaccurate (6). The Global Leadership Initiative on Malnutrition (GLIM) Criteria provided a standardized approach to diagnose malnutrition in 2018 (7), and GLIM-defined malnutrition has been widely used and has been well recognized as a poor prognostic indicator for clinical outcomes in patients with cancer (8, 9). The previous studies mainly focused on preoperative risk assessment such as malnutrition, obesity, low muscle mass, preoperative comorbidities, etc. (2, 10). However, ΔAlb as a composite biomarker reflecting the intraoperative state had never been used in combination with preoperative risk factors for the prediction of postoperative outcomes.

Therefore, the objective of this study was aimed to explore the relationship between ΔAlb and malnutrition, and investigate the prognostic value of ΔAlb for the prediction of postoperative outcomes in rectal cancer patients with normal preoperative albumin levels, when in combination with malnutrition. We also aimed to investigate the possible factors associated with ΔAlb and provide assistance for the perioperative management.

## 2 Materials and methods

### 2.1 Patients

From January 2013 to April 2019, all patients who underwent surgery for rectal cancer at the Department of Surgery, The Second Affiliated Hospital of Wenzhou Medical University were included in this study. The inclusion criteria included patients who (i) were ≥ 18 years; (ii) planned to receive elective surgery for rectal cancer with curative intent; and (iii) had abdominal computed tomography (CT) scans available for review within 1 month before surgery. Exclusion criteria included (i) those undergoing palliative surgery or emergency surgery; (ii) those receiving neo-adjuvant treatment; (iii) those treated with exogenous albumin preoperatively or on the first postoperative day; and (iv) those with severe organ dysfunction (kidney, liver, or heart) or incomplete laboratory data. The surgical procedures were performed by surgeons with extensive experience according to the Colorectal Cancer Treatment Guidelines. The routine postoperative management comprised the following: laboratory tests on the first day after surgery and every 3 days, administration of preventive antibiotics, enteral or parenteral intervention, and albumin infusion was recommended for patients with severe hypoalbuminemia (albumin levels < 30 g/L) or hypovolemia. The data collection protocol for this study was approved by the Ethics Committee of the Second Affiliated Hospital of Wenzhou Medical University (LCKY2020–209).

### 2.2 Data collection

The following data were collected: (i) general features, including age, gender, BMI, preoperative hemoglobin, preoperative and postoperative albumin, skeletal muscle index (SMI), Charlson comorbidity index (CCI) (11), American Society of Anesthesiology (ASA) grade, nutritional status, previous abdominal surgery; (ii) the tumor characteristics and operative details, tumor location, pathological tumor node metastasis (TNM) stage, type of surgery, laparoscopic-assisted surgery, and surgical duration, intraoperative fluid use and estimated blood loss; (iii) postoperative outcomes, postoperative complication, postoperative hospital stays.

### 2.3 Definitions

Plasma albumin levels < 35 g/L were defined as hypoalbuminemia. Hemoglobin levels < 120 g/L for men or < 110 g/L for women were defined as anemia. ∆ALB was defined as follows: (preoperative albumin-postoperative albumin on the first postoperative day)/ preoperative albumin×100%. Malnutrition was diagnosed using a two-step approach according to the GLIM consensus criteria (7). First, Nutritional Risk Screening 2002 was applied to identify the individuals at risk of malnutrition. Second, since patients with cancer had already fulfilled one of the etiological criteria (burden of disease), malnutrition was defined if one of the three phenotypical criteria was satisfied. (i) weight loss: nonvoluntary weight loss of more than 5% within the previous 6 months or more than 10% of any time; (ii) low BMI: BMI of less than 20 kg/m2 for patients older than 70 years or less than 18.5 kg/m2 for those younger than 70 years; and (iii) low muscle mass: assessed by SMI based on the preoperative abdominal CT images at the level of the third lumbar vertebra. As previously described, low SMI were identified as < 40.8 cm2 /m2 for males or < 34.9 cm2 /m2 for females (12). The cut-off values for surgical duration (13), intraoperative fluid use and estimated blood loss were established according to the upper quartile or previous report. Complications within 30 days after surgery were calculated and stratified using the Clavien-Dindo (CD) classification (14). Complications classified as grade II or above were analyzed, and complications classified as grade III or higher were considered severe postoperative complications.

### 2.4 Statistical analyses

Continuous variables were expressed as the mean and standard deviation (SD) or median and interquartile ranges (IQR). Categorical variables were expressed as numbers and percentages. Differences between groups were analyzed using Student’s t test, Pearson’s chi-square test, Fisher’s exact test or the Mann–Whitney U test as appropriate. Receiver operating characteristic (ROC) curve analysis was used to determine a cutoff for ΔAlb associated with postoperative complications. Variables with a significant trend (*p* < 0.1) in the univariate analysis, were included in the multivariate forward logistic regression analysis. Statistical significance was defined as a *p* < 0.05. All data were analyzed using SPSS statistics version 22.0 (IBM, Armonk, New York, USA).

## 3 Results

### 3.1 Cutoff value for ΔAlb

**Figure 1:**
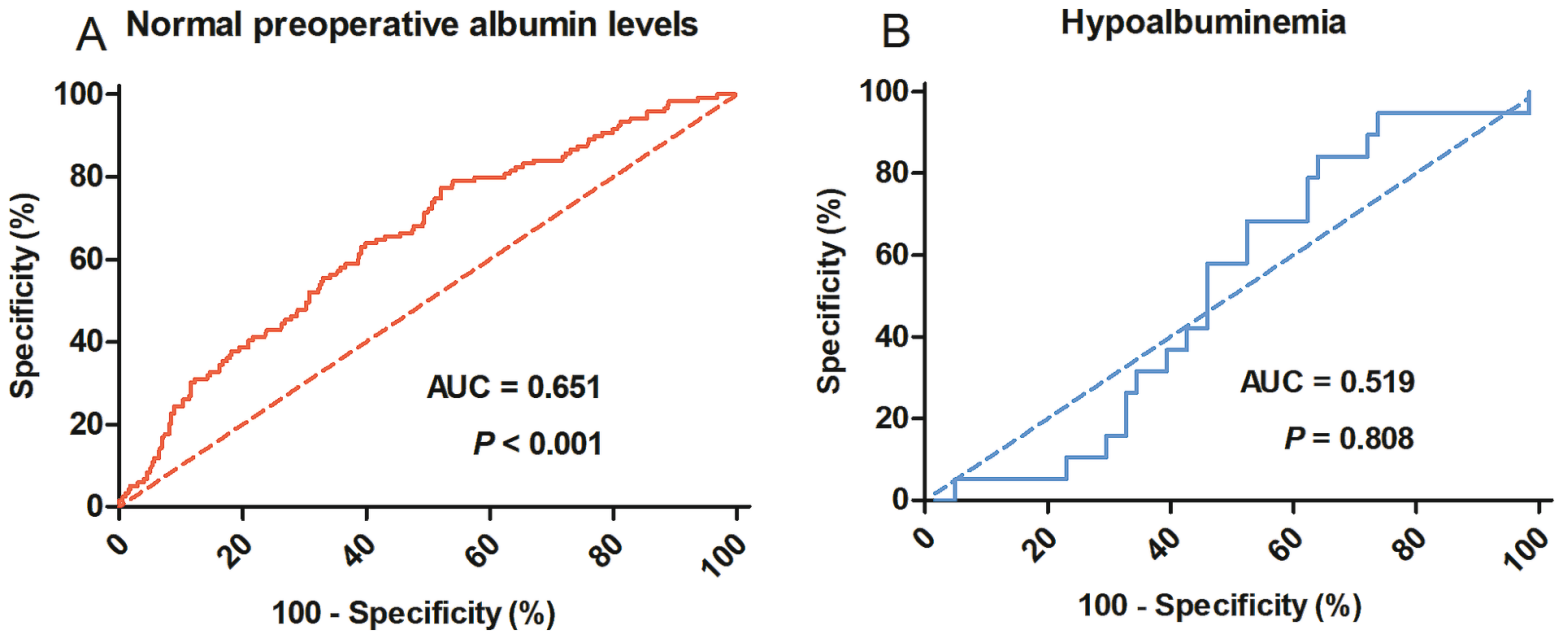
Receiver operating characteristic curves to identify postoperative complications in rectal cancer patients with normal preoperative albumin levels **(A)** or hypoalbuminemia **(B)**. AUC, Area under the curve.

From January 2013 to August 2019, a total of 526 patients who met the inclusion and exclusion criteria were enrolled in this study. As shown in Figure 1, the predictive value of ∆Alb for postoperative complications was evaluated by ROC curve analysis. ∆Alb was significantly associated with postoperative complications in patients with normal preoperative albumin levels (*p* < 0.001), but was not in patients with hypoalbuminemia (*p* = 0.808). The optimal cut-off value was calculated at 15.86% (16% was applied in the following) and the area under the curve (AUC) was 0.651 (95% confidence interval 0.596–0.706) (Figure 1A).

### 3.2 Clinicopathological characteristics

Patient clinicopathologic characteristics were summarized in Table 1. Based on the cut-off value of 16%, 301 patients (57.2%) were categorized into the ΔAlb ≥ 16% group, while the remaining 225 patients (42.8%) were categorized into the ∆Alb < 16% group. There were no significant differences in age, CCI, ASA grade, previous abdominal surgery, laparoscopic-assisted surgery, TNM stage and estimated blood loss between ΔAlb ≥ 16% and ∆Alb < 16% groups. Patients with ΔAlb ≥ 16% were more likely to be female (*p* = 0.033), had higher preoperative hemoglobin (*p* = 0.019) and albumin levels (*p* < 0.001), and more prevalence of malnutrition (*p* = 0.03), but lower BMI (*p* = 0.002) and skeletal muscle index (SMI) (*p* < 0.001) compared to those with ∆Alb < 16%. Tumor locations were significantly lower (*p* < 0.001) in individuals with ΔAlb ≥ 16%, and there was a greater frequency of Miles surgery or enterostomy (*p* < 0.001), accompanied with longer surgical duration (*p* = 0.02) and more intraoperative fluid use (*p* = 0.001).

**Table 1:** Patient baseline characteristics.

Factors	All (*n* = 526)	ΔAlb<16% (*n* = 225)	ΔAlb≥16% (*n* = 301)	Factors
Age, mean (SD), years	63.98 ± 11.74	63.55 ± 10.98	64.3 ± 12.28	0.470
Gender				0.033^*^
Female	210 (39.9)	78 (34.7)	132 (43.9)	
Male	316 (60.7)	147 (65.3)	169 (56.1)	
Preoperative Hb, mean, (SD), g/L	129.99 ± 16.47	128.04 ± 17.20	131.45 ± 15.78	0.019^*^
Preoperative Alb, median, (IQR), g/L	39.95 (4.2)	39 (3.75)	40 (4.3)	<0.001^*^
BMI, mean (SD),kg/m2	22.67 ± 3.22	23.16 ± 3.15	22.31 ± 3.24	0.002^*^
CCI				0.511
0	457 (86.9)	198 (88.0)	259 (86.0)	
≥1	69 (13.1)	27 (12.0)	42 (14.0)	
ASA grade				0.283
I/II	458 (87.1)	200 (88.9)	258 (85.7)	
III	68 (12.9)	25 (11.1)	43 (14.3)	
SMI, mean (SD), cm2/m2	42.93 ± 8.45	44.69 ± 8.24	41.61 ± 8.39	<0.001^*^
GLIM-defined malnutrition				0.030^*^
No	406 (77.2)	184 (81.8)	222 (73.8)	
Yes	120 (22.8)	41 (18.2)	79 (26.2)	
Previous abdominal surgery				0.943
No	474 (90.1)	203 (90.2)	271 (90.0)	
Yes	52 (9.9)	22 (9.8)	30 (10.0)	
Tumor location				<0.001^*^
Low third	148 (28.1)	36 (16.0)	112 (37.2)	
Middle third	230 (43.7)	114 (50.7)	116 (38.5)	
High third	148 (28.1)	75 (33.3)	73 (24.3)	
Type of surgery				<0.001^*^
Miles	83 (15.8)	16 (7.1)	67 (22.3)	
Hartmann	6 (1.1)	1 (0.4)	5 (1.7)	
Dixon+Enterostomy	50 (9.5)	16 (7.1)	34 (11.3)	
Dixon	387 (73.6)	192 (85.3)	195 (64.8)	
Laparoscopic-assisted surgery				0.329
No	415 (78.9)	173 (76.9)	242 (80.4)	
Yes	111 (21.1)	52 (23.1)	59 (19.6)	
TNM stage				0.393
Tis/T1	143 (27.2)	65 (28.9)	78 (25.9)	
T2	157 (29.8)	71 (31.6)	86 (28.6)	
T3	226 (43.0)	89 (39.6)	137 (45.5)	
Surgical duration, mean, (SD), min	160 (70)	155 (65)	165 (70)	0.020^*^
Estimated blood loss, median (IQR), ml	150 (128.75)	150 (140)	150 (101.5)	0.205
Intraoperative fluid use, median (IQR), ml	2,500 (1000)	2,500 (1000)	2,600 (1050)	0.001^*^

Values in parentheses are percentages unless indicated otherwise. *Statistically significant (*p* < 0.05).

IQR, interquartile range; SD, standard deviation; HB, hemoglobin; SMI, skeletal muscle index; BMI, body mass index; CCI, Charlson Comorbidity Index; ASA, American Society of Anesthesiologists; TNM, Tumor-Node-Metastasis.

### 3.3 Short-term surgical outcomes

As demonstrated in Table 2, a total of 119 patients (22.6%) experienced postoperative complications. ΔAlb ≥ 16% and malnutrition alone significantly increased the incidence of postoperative complications (29.6%, *p* < 0.001 and 30.8%, *p* = 0.014 respectively), and it was raised to 38% (*p* < 0.001) when taken then together. Detail analysis of the complications showed that malnutrition mainly influenced medical complications (*p* = 0.005), while ∆ALB ≥ 16% influenced both surgical (*p* < 0.001) and medical (*p* = 0.035) complications. ΔAlb ≥ 16% had significantly prolonged postoperative hospital stays (*p* < 0.001), whereas malnutrition did not (*p* = 0.408).

**Table 2:** Postoperative outcomes.

Outcomes	Overall (*n* = 526)	∆ALB (≥16%) (*n* = 301)	*^c^p*	Malnutrition (*n* = 120)	*^c^p*	∆ALB (≥16%) + Malnutrition (*n* = 79)	*^c^P*
^a^Total complications	119 (22.6)	89 (29.6)	<0.001^*^	37 (30.8)	0.014	30 (38.0)	< 0.001^*^
^b^Severe complications	28 (5.3)	22 (7.3)	0.019	6 (5.0)	0.858	5 (6.3)	0.293
Detail of complications							
Surgical complications	70 (13.3)	54 (17.9)	<0.001^*^	18 (15.0)	0.535	14 (17.7)	0.005^*^
Wound infection	16 (3)	13 (4.3)	0.049*	6 (5.0)	0.263	4 (5.1)	0.049*
Intestinal obstruction	11 (2.1)	8 (2.7)	0.458	5 (4.2)	0.148	4 (5.1)	0.126
Intra-abdominal infection	10 (1.9)	8 (2.7)	0.251	1 (0.8)	0.552	1 (1.3)	0.611
Anastomotic leakage	9 (1.7)	5 (1.7)	0.812	1 (0.8)	0.658	1 (1.3)	0.999
Blood transfusion	9 (1.7)	7 (2.3)	0.359	4 (3.3)	0.246	3 (3.8)	0.154
Bleeding	8 (1.5)	7 (2.3)	0.166	0 (0)	0.261	0 (0)	0.663
Anterior resection syndrome	5 (1.0)	4 (1.3)	0.562	1 (0.8)	0.700	1 (1.3)	0.876
Ureteral fistula	2 (0.4)	2 (0.7)	0.611	0 (0)	0.941	0 (0)	/
Medical complications	49 (9.3)	35 (11.6)	0.035*	19 (15.8)	0.005*	16 (20.3)	< 0.001^*^
Urinary infection	17 (3.2)	13 (4.3)	0.103	8 (6.7)	0.033*	6 (7.6)	0.015
Pulmonary complications	12 (2.3)	8 (2.7)	0.504	2 (1.7)	0.869	1 (1.3)	0.743
Hyperthermia	8 (1.5)	5 (1.7)	0.955	4 (3.3)	0.155	4 (5.1)	0.243
Venous thrombosis	5 (1.0)	3 (1.0)	0.743	0 (0)	0.493	0 (0)	0.876
Cardiac complications	4 (0.8)	4 (1.3)	0.219	4 (3.3)	0.002*	4 (5.1)	0.012*
Urinary retention	2 (0.4)	1 (0.3)	0.611	0 (0)	0.941	0 (0)	0.663
Cerebral infarction	1 (0.2)	1 (0.3)	0.884	1 (0.8)	0.517	1 (1.3)	0.663
Postoperative hospital stays, median (IQR), days	16 (4.48)	17 (6.59)	<0.001*	16 (6)	0.408	17 (7.45)	0.001*

Values in parentheses are percentages unless indicated otherwise.

^a^Complications classified as grade II and above. ^b^Complications classified as grade III and above. ^c^Compared with the opposite group.

^*^Statistically significant (*p* < 0.05).

In the univariate analysis (Table 3), postoperative complications were linked with malnutrition (*p* = 0.014), ∆Alb (*p* < 0.001), tumor location (*p* < 0.001), type of surgery (*p* < 0.001), surgical duration (*p* = 0.002) and estimated blood loss (*p* = 0.032). In the multivariate logistic regression analysis, malnutrition (OR 1.730, 95% CI 1.073–2.789, *p* = 0.024), ∆Alb ≥ 16% (OR 2.179, 95% CI 1.354–3.506, *p* < 0.001), tumor located in the lower third (OR 2.370, 95% CI 1.319–4.258, *p* = 0.004) and surgical duration ≥ 180 min (OR 1.699, 95% CI 1.100–2.625, *p* = 0.017) were identified as independent risk factors for postoperative complications in rectal cancer surgery.

**Table 3:** Univariate and multivariate analysis of risk factors for postoperative complications.

Factors	Univariate analysis			Multivariate analysis	
	Complication (%)	OR (95% CI)	*p*	OR (95% CI)	*p*
Age			0.992		
≥65/<65	59 (22.6)/60 (22.6)	0.998 (0.663–1.501)			
Gender			0.917		
Male/Female	71 (22.5)/48 (22.9)	0.978 (0.645–1.483)			
Anemia			0.811		
Yes/No	18 (23.7)/101 (22.4)	1.072 (0.604–1.903)			
BMI			0.074		
< 18.5	18 (35.3)	1.989 (1.052–3.759)			
18.5–24	65 (21.5)	1			
˃ 24	36 (20.8)	0.958 (0.606–1.515)			
Charlson comorbidity index			0.096		
≥ 2/< 2	21 (30.4)/98 (21.4)	1.603 (0.916–2.804)			
ASA grade			0.152		
III/I,II	20 (29.4)/99 (21.6)	1.511 (0.857–2.664)			
Low muscle mass			0.609		
Yes/No	35 (24.1)/84 (22.0)	1.125 (0.717–1.766)			
Previous abdominal surgery			0.259		
Yes/No	15 (28.8)/104 (21.9)	1.442 (0.762–2.730)			
GLIM-defined malnutrition			0.014*	1.730 (1.073–2.789)	0.024*
Yes/No	37 (30.8)/82 (20.2)	1.761 (1.115–2.782)			
∆ALB (≥16%)			<0.001*	2.179 (1.354–3.506)	0.001*
Yes/No	89 (29.6)/30 (13.3)	2.729 (1.727–4.310)			
^a^∆ALB combine with malnutrition			<0.001*	3.779 (1.981–7.207)	<0.001*
Yes/Both No	30 (38.0)/23 (12.5)	4.286 (2.282–8.050)			
Tumor location			<0.001*		
Low third	51 (34.5)	3.011 (1.710–5.302)		2.370 (1.319–4.258)	0.004*
Middle third	46 (20.0)	1.432 (0.821–2.497)		1.445 (0.817–2.553)	0.206
High third	22 (14.9)	1		1	
Type of surgery			<0.001*		
Miles	33 (39.8)	2.989 (1.795–4.978)			
Hartmann	2 (33.3)	2.264 (0.407–12.607)			
Dixon+Enterostomy	14 (28.0)	1.761 (0.902–3.440)			
Dixon	70 (18.1)	1			
laparoscopic-assisted surgery			0.821		
Yes/No	26 (23.4)/93 (22.4)	1.059 (0.645–1.739)			
TNM stage			0.363		
Tis, I	27 (18.9)	1			
II	35 (22.3)	1.233 (0.702–2.164)			
III	57 (25.2)	1.449 (0.866–2.426)			
Surgical duration (≥180 min)			0.002*	1.699 (1.100–2.625)	0.017*
Yes/No	58 (30.2)/61 (18.3)	1.937 (1.280–2.933)			
Estimated blood loss (≥200 mL)			0.032*		
Yes/No	65 (26.9)/54 (19.0)	1.564 (1.037–2.358)			
Intraoperative fluid use (≥3,000 mL)			0.167		
Yes/No	54 (25.7)/65 (20.6)	1.337 (0.885–2.019)			

^*^Statistically significant (*p* < 0.05). OR, Odds Ratio; CI, Confidence Interval.

^a^Compared with the opposite group (ΔAlb <16% and without malnutrition).

### 3.4 Factors associated with ∆ALB

As shown in Table 4, univariate analysis revealed that gender, BMI, low muscle mass, malnutrition, tumor location, type of surgery, surgical duration and intraoperative fluid use were significantly associated with ∆ALB. In the multivariate analysis, low muscle mass (OR = 2.058, 95% CI 1.351–3.135, *p* < 0.001), tumor located in the lower third (OR = 2.909, 95% CI 1.757–4.818, *p* < 0.001) and surgical duration ≥ 180 min (OR = 1.659, 95% CI 1.129–2.439, *p* = 0.01) were identified as independent risk factors associated with ∆ALB.

**Table 4:** Univariate and multivariate analysis of risk factors for postoperative ∆ALB ≥16%.

Factors	Univariate analysis			Multivariate analysis	
	Complication (%)	OR (95% CI)	*p*	OR (95% CI)	*p*
Age			0.950		
≥65/<65	149 (57.1)/152 (57.4)	0.989 (0.700–1.397)			
Gender			0.033*		
Male/Female	169 (53.5)/132 (62.9)	0.679 (0.476–0.970)			
Anemia			0.104		
Yes/No	37 (48.7)/264 (58.7)	0.668 (0.411–1.088)			
BMI			0.005*		
< 18.5	39 (76.5)	2.391 (1.204–4.748)			
18.5–24	174 (57.6)	1			
˃ 24	88 (50.9)	0.762 (0.523–1.109)			
Charlson comorbidity index			0.511		
≥ 2/< 2	42 (60.9)/259 (56.7)	1.189 (0.709–1.996)			
ASA grade			0.283		
III/I,II	43 (63.2)/258 (56.3)	1.333 (0.788–2.257)			
Low muscle mass			<0.001*	2.058 (1.351–3.135)	<0.001*
Yes/No	100 (69.0)/201 (52.8)	1.990 (1.327–2.984)			
GLIM-defined malnutrition			0.030*		
Yes/No	79 (65.8)/222 (54.7)	1.597 (1.044–2.442)			
Previous abdominal surgery			0.943		
Yes/No	30 (57.7)/271 (57.2)	1.021 (0.572–1.823)			
Tumor location			<0.001*		
Low third	112 (75.7)	3.196 (1.949–5.243)		2.909 (1.757–4.818)	<0.001*
Middle third	116 (50.4)	1.045 (0.692–1.580)		1.030 (0.676–1.568)	0.891
High third	73 (49.3)	1			
Type of surgery			<0.001*		
Miles	67 (80.7)	4.123 (2.307–7.369)			
Hartmann	5 (83.3)	4.923 (0.570–42.529)			
Dixon+Enterostomy	34 (68.0)	2.092 (1.118–3.916)			
Dixon	195 (50.4)	1			
laparoscopic-assisted surgery			0.329		
Yes/No	59 (53.2)/242 (58.3)	0.811 (0.533–1.235)			
TNM stage			0.393		
Tis, I	78 (54.5)	1			
II	86 (54.8)	1.009 (0.640–1.591)			
III	137 (60.6)	1.283 (0.840–1.960)			
Surgical duration (≥180 min)			0.003*	1.659 (1.129–2.439)	0.01
Yes/No	126 (65.6)/175 (52.4)	1.735 (1.202–2.504)			
Estimated blood loss (≥200 mL)			0.184		
Yes/No	146 (60.3)/155 (54.6)	1.266 (0.894–1.792)			
Intraoperative fluid use (≥3,000 mL)			0.008*		
Yes/No	135 (64.3)/166 (52.5)	1.627 (1.137–2.328)			

^*^Statistically significant (*p* < 0.05). OR, Odds Ratio; CI, Confidence Interval.

## 4 Discussion

Although ∆ALB has been widely acknowledged as a negative prognostic marker for clinical outcomes after major abdominal surgery, several crucial matters must be pointed out. Firstly, the extent of postoperative albumin reduction would be influenced by the preoperative baseline level, hence ∆ALB could not accurately represent the severity of surgical trauma in patients with hypoalbuminemia, which had been neglected in most previous studies (13, 15–19) and had been confirmed in our study. The present study showed that ∆ALB was significantly associated with postoperative complications in patients with normal preoperative albumin levels, whereas no such association was observed in patients with hypoproteinemia. Secondly, as the primary outcome, the definitions of postoperative complications analyzed in the previous studies were various, including overall complications (CD grade ≥ I) (13, 16–18, 20), major complications (CD grade ≥ III) (15, 19) or infectious complications (21). Actually, it is more meaningful to investigate complications classified as grade II or above and which had been widely used in the previous studies (10, 22). In the current study, complications CD grade ≥ II were analyzed and the cutoff value of ∆ALB was established at 16%, which significantly distinguished patients at low and high risk for postoperative complications. Thirdly, the previous research included various types of abdominal surgery and the specificity of certain surgeries may influence the pathophysiology of albumin levels.

Albumin is widely used in clinical practice as a convenient indicator of nutritional status, although it was considered to be inaccurate (6, 20). Hypoproteinemia is often treated with special attention, while not for patients with normal preoperative serum albumin which has usually been considered in normal nutritional status. The GLIM criteria have been increasingly recognized as an effective tool for nutritional assessment recently (8, 9). In the present study, we found a high prevalence (22.81%) of GLIM-defined malnutrition among patients with normal preoperative serum albumin. The multivariate analysis showed that ∆ALB ≥ 16% and malnutrition were both independent risk factors for postoperative complications with an OR of 2.179 and 1.730, respectively. When combined together, the OR would reach to 3.779. Malnutrition primarily influenced medical complications, while ∆ALB ≥ 16% was correlated with both surgical (*p* < 0.001) and medical (*p* = 0.035) complications (mainly the surgical complications). Therefore, malnutrition, a preoperative risk indicator and ∆ALB ≥ 16%, an intraoperative risk indicator, should be combined to enhance the predictive value for postoperative outcomes. And it is necessary to conduct a nutritional assessment for patients with normal postoperative albumin levels to distinguish patients with malnutrition.

The possible reasons for rapid decline in albumin levels following surgery are primarily due to capillary leak induced by the inflammatory response to the surgical trauma, along with decreased hepatic production and dilution of serum albumin (4). It is reported that low level of albumin was related to malnutrition (6), however the relationship between ΔAlb and malnutrition remains unclear. In the present study, malnutrition had a significant impact on ΔAlb, but was supplanted by low muscle mass which emerged as an independent risk factor in the multivariate analysis. The possible reason may be that low muscle mass has a direct effect on protein metabolism, as the mobilization of muscle proteins would provide free amino acids that are used for energetic purpose and the synthesis of proteins (23), which maybe blocked in patients with low muscle mass. It is interesting that gender male was identified as a protective factor for ΔAlb, but not an independent risk factor, which may be explained by the muscle mass because the men are naturally equipped with a greater amount of muscle mass compared to women, and Labgaa et al. also detected this phenomenon (16, 19). We also identified tumor location and surgical duration as independent risk factors for ΔAlb, which was understandable. Tumors situated at lower positions present a greater challenge during surgery, resulting in longer operative duration and increased surgical stress, which can subsequently lead to a significant reduction in postoperative albumin levels. Consistent with previous studies, intraoperative fluid use was associated with ΔAlb, but was not an independent risk factor, which should be adjusted by surgical duration in the multivariate regression model. In short, ΔAlb serves as a meaningful indicator, not only mirroring the surgical stress response in a certain extent, but also signifying the patient’s capacity to withstand stress. Therefore, the assessment of ΔAlb is strongly advised for stratifying patients with higher risk of developing postoperative complications, especially for the rectal cancer patients with normal preoperative albumin levels.

Is it beneficial to mitigate ΔAlb with albumin supplementation? Most previous studies hold a negative view (24, 25). On the contrary, the use of exogenous albumin may lead to increased albumin leakage, heightened risks of swelling, and other related complications (24). Recently, a randomized clinical trial (5) concluded that goal-directed albumin substitution in a surgical population with hypoalbuminemia < 30 g/L did not reduce the incidence of postoperative complications and suggested that previously identified advantages of albumin supplementation on renal function (26) were found to be temporary. Instead of albumin supplementation, enhanced recovery programmes (ERAS) and nutritional intervention are recommended to attenuate the surgical stress and systemic inflammation, avoid perioperative fluid overload and maintain nutrient supply (4, 16). However, considering the physiologic functions of serum albumin, exogenous albumin is recommended for use by the Practice Guideline (27) when serum albumin < 20 g/L after normalization of circulatory volume.

The current study had several limitations. First, although we endeavored to adjust the impact of confounding factors as many as possible, the retrospective design of our study carried a substantial risk of selection bias. Secondly, as a single-center study, perioperative management strategies were based on our local experience. The findings of this study need to be confirmed in multicenter prospective studies in the future. Thirdly, due to the absence of a standardized threshold for low SMI in patients with colorectal cancer, we adopted a commonly utilized value for SMI from a previous study (12).

## 5 Conclusion

The present study demonstrated that ∆ALB had significantly predictive value for postoperative outcomes in rectal cancer patients with normal preoperative albumin levels, but not for the patients with hypoproteinemia. And, ΔAlb in combination with GLIM-defined malnutrition would obviously enhance the predictive value for postoperative outcomes. According to our findings, more attention should be paid to patients with normal preoperative albumin levels, ∆ALB and nutritional assessments were highly recommended to provide information for risk stratification, prognosis prediction and decision making.

## Data Availability

The original contributions presented in the study are included in the article/supplementary material, further inquiries can be directed to the corresponding authors.
